# Role of the *Aspergillus*-Specific IgG and IgM Test in the Diagnosis and Follow-Up of Chronic Pulmonary Aspergillosis

**DOI:** 10.3389/fmicb.2019.01438

**Published:** 2019-06-25

**Authors:** Hongxing Li, Yuwen Rui, Wei Zhou, Lulu Liu, Binchan He, Yi Shi, Xin Su

**Affiliations:** ^1^Department of Respiratory and Critical Care Medicine, Jinling Hospital, Southern Medical University, Nanjing, China; ^2^Department of Respiratory and Critical Care Medicine, Jinling Hospital, Nanjing University Medical School, Nanjing, China

**Keywords:** *Aspergillus*-specific IgG, *Aspergillus*-specific IgM, chronic pulmonary aspergillosis, subacute invasive aspergillosis, diagnosis

## Abstract

**Background:**

Chronic pulmonary aspergillosis (CPA) has a high rate of misdiagnosis and has been reported to have an increasing rate of morbidity and mortality. In this article, we assessed the serum *Aspergillus*-specific IgG and IgM test in the diagnosis of patients with CPA.

**Methods:**

A prospective study was conducted from January 2016 to July 2017 in Nanjing Jinling Hospital. Serum samples were collected from CPA patients (178 sera, 82 patients) and from non-aspergillosis patients (125 sera) with community-acquired pneumonia (CAP), active tuberculosis, bronchiectasis or lung tumors. Additionally, we included a control group of healthy patients(50 sera). *Aspergillus*-specific antibody detection was performed using a Dynamiker ELISA kit, and the results were compared with the value of galactomannan (GM) in bronchoalveolar lavage fluid (BALF).

**Results:**

The sensitivity and specificity of the *Aspergillus*-specific IgG antibody in the diagnosis of CPA were 84.1 and 89.6%, respectively. These values were slightly higher compared to those obtained for the sensitivity and specificity using the BALF GM test (79.1 and 84.2%, respectively). However, the sensitivity and specificity of *Aspergillus*-specific IgM antibody were only 43.9 and 87.2%, respectively. Moreover, the positive rate of IgG in patients with subacute invasive aspergillosis (SAIA) was 87%, compared to the positive rates of IgG in CPA patients sick for 3–6 months (80.0%), 6–9 months (81.8%) and ≥9 months (80.0%). Meanwhile, the positive rate of IgM in SAIA patients was 63%, compared to the positive rate of IgM in CPA patients sick for 3–6 months (46.7%), 6–9 months (0%) and ≥9 months (0%), respectively. Furthermore, serum IgG levels decreased gradually in the majority of CPA patients who showed positive response to antifungal therapy, and IgG levels increased in two CPA patients when their disease worsened.

**Conclusion:**

A serum *Aspergillus*-specific IgG test is a valuable tool for the diagnosis of CPA and SAIA, while an *Aspergillus*-specific IgM test is only modestly specific for the diagnosis of SAIA. Overall, the variation trend of *Aspergillus*-specific IgG levels may reflect the therapeutic effectiveness in the long-term follow-up of CPA.

## Introduction

Chronic pulmonary aspergillosis has been increasingly reported in patients with underlying respiratory disorders, such as COPD, cavitary pulmonary tuberculosis, and bronchiectasis, and in patients given glucocorticoids or immunosuppressants (Garnacho-Montero et al., 2005; Wichmann and Kluge, 2013; Denning et al., 2015; Yan et al., 2016). Because of the relatively intact immune system, these patients often display atypical symptoms coupled with non-standard imaging results, making the early diagnosis difficult. For this reason, many diagnostic methods have been developed to identify fungal infection including biopsy collection or cell culture, detection of the fungal cell wall component, polymerase chain reaction for fungal genes and antibody detection. Among these methods, antibody detection has been considered the most effective CPA screening strategy. Although a few studies discuss the diagnostic value of the As-IgG antibody detection approach (Baxter et al., 2013; Denning et al., 2015; Dumollard et al., 2016; Page et al., 2016), they focus on a small sample number and make no additional analyses or association in regards to the disease course in patients. The levels of As-IgA and As-IgM were reported as elevated in some CPA cases (Kauffman et al., 1986), but previous studies were not very comprehensive and were characterized by very low sensitivity and specificity limiting their application (Schonheyder and Andersen, 1982; Weig et al., 2001; Du et al., 2012). Moreover, previous studies did not address whether As-IgG and As-IgM could be used to monitor the treatment response in patients with CPA. In this study, we explored the diagnostic value of As-IgG and As-IgM using the Dynamiker assay using CPA samples and examined the results in the context of treatment response monitoring.

## Materials and Methods

This was a prospective study conducted between January 2016 and July 2017 at the Department of Respiratory and Critical Care Medicine, Nanjing Jinling Hospital. Diagnosis was made by senior clinicians and was validated by authors based on the published guidelines. The study protocol was approved by the Institute Ethics Committee of Jinling Hospital (2015NJKY-035-02) and written informed consent was obtained from all patients.

### Study Patients

Following exclusion of cases with no definitive diagnosis, 353 sera from 257 subjects were collected and divided into the CPA patient group (*n* = 82 patients with 34 patients with *proven* diagnosis and 48 patients with *probable* diagnosis) with a total of 178 sera samples and non-CPA group patients (*n* = 125 patients diagnosed with CAP, active tuberculosis, bronchiectasis or lung tumors) with a total of 125 sera samples. Finally, we also included 50 healthy control patients with 50 sera samples. All samples were stored frozen at –80°C before analysis. Moreover, we collected 56 BALF GM data points from the CPA group patients and 63 BALF GM results from the non-CPA group.

### Criteria for CPA Diagnosis

This diagnosis was based on the updated criteria of European Society of Clinical Microbiology and Infectious Diseases and European respiratory association (ESCMID and ERS) guidelines for the management of CPA and the updated Infectious disease society of America (IDSA) guidelines (Denning et al., 2015; Patterson et al., 2016). The diagnosis of CPA included the following criteria:(1) over 3 months of pulmonary symptoms or chronic illness such as cough, sputum, hemoptysis, dyspnea and other clinical symptoms of lower respiratory tract infection; (2) persistent, stable or slowly progressive radiographic abnormalities consistent with pulmonary aspergillosis, such as consolidation, or nodules with or without cavitation as per thoracic imaging; (3) positive microbiological results, such as positive sputum or BALF culture of *Aspergillus spp*, positive BALF GM or positive serum GM. Positive antibody test results were not used in the diagnosis; (4) histological or cytopathological evidence indicating *Aspergillus*-like hyphae present in lesions from lung biopsies or lobectomy, or positive cultures from sterile samples (e.g., aspiration fluid from percutaneous lung biopsies). A *proven* diagnosis was made based on all four criteria, while *probable* diagnosis was made based on the first three criteria. The diagnosis criteria of SAIA was consist of (1) more rapidly progressive clinical symptoms and radiological changes (usually within a period of 3 months); (2) with some host factors such as COPD, diabetes, cavitary pulmonary tuberculosis or glucocorticoid or immunosuppressant usage history; (3)microbiological evidence of positive serum or BALF GM or positive culture results.

### As-IgG and As-IgM Antibody Tests

*Aspergillus*-specific As-IgG and As-IgM levels were measured using the *Aspergillus fumigates* IgG and IgM Quantitative Test kit (Dynamiker, China), based on enzyme-linked immunosorbent assay (ELISA), according to the manufacturers’ instructions. Briefly, serum samples were diluted 1:1000 and incubated in ELISA plates for 60 min at 37°C. After 3 rounds of washing, an enzyme-labeled anti-human IgG was added and incubated for 30 min at 37°C. Following additional 3 washing steps, substrate solution was added and the mixture was incubated for 15 min at 37°C in the dark. The reaction was stopped by the addition of stop buffer and the OD was measured at 450 nm using a plate reader. Values ≥120 AU/ml were considered positive, values <80 AU/ml were considered negative and values of 80–120 AU/ml were classified as borderline. These borderline values were evaluated in the context of clinical and radiological features and in response to antifungal therapy. This antibody ELISA assay relies on a recombinant antigen extracted from various *Aspergillus fumigates* strains. Therefore, it is important to consider that this recombinant *Aspergillus* antigen could be used to detect different kinds of *Aspergillus* infections.

### Bronchoalveolar Lavage Fluid (BALF) and Serum GM Test

Bronchoalveolar lavage fluid and serum GM values were measured in the clinical laboratory using “double-sandwich” ELISA according to the manufacturer’s instructions with the Platelia TM Aspergillus Kit (Bio-Rad Laboratories, Hercules, CA, United States). We set the optimal cut-off OD index for BALF GM at 0.7 based on our previous study (Zhou et al., 2017). The index value was set to 0.5 for serum GM according to the manufacturer’s instructions.

Each patient had more than one serum sample. Because the levels of As-IgG and As-IgM in CPA patients may decrease after treatment, sensitivities and specificities were calculated based on the sample collected prior to the antifungal treatment.

Status of patients with CPA was considered improved once the initial clinical manifestations completely disappeared, alleviated or became stable and pulmonary lesions shrank, as indicated by imaging examination. The status of CPA patients was labeled as progressive if original clinical manifestations worsened, image examination showed exacerbated lesions or if new symptoms appeared (Herbrecht et al., 2002; Jhun et al., 2013).

### Statistical Methods

Software SPSS 20.0 was used to analyze quantitative data. Fisher’s exact test and Wilcoxon test were used to compare the baseline characteristics of the groups of patients. Independent sample *t*-test was used to analyze the differences between the CPA group and non-CPA group. Sensitivity, specificity, PPV and NPV were calculated to evaluate the diagnostic value of serum As-IgG and As-IgM test and BALF GM test in patients with CPA. *P* < 0.05 was considered statistically significant. The false positive rate was defined as the relative frequency of no disease when the disease was predicted. The false negative rate was defined as the relative frequency of disease occurring when no disease was predicted.

## Results

### Patient Characteristics

The baseline characteristics of the study population are shown in Table 1. The mean age of the CPA group, non-CPA group and healthy control group was 58 years (range 16–88), 55 years (range 14–84) and 56 years (range 26–72), respectively. Higher number of patients in the CPA group suffered from COPD compared to the non-CPA group and this difference was statistically significant (*P* < 0.01). Moreover, patients in the CPA group used more corticosteroids than patients in the non-CPA group (*P* < 0.01) (Table 1).

**TABLE 1:** Characteristics of 82 Patients with CPA and 125 without CPA.

**Index**	**CPA (*n* = 82)**	**non-CPA (*n* = 125)**	***P*-value**
Proven; probable	34;48	–	–
Sex (male/female)	58/24	78/47	0.069
Age (median years, range)	58 (16–88)	55 (14–84)	0.154
**Underling pulmonary diseases**			
COPD	29 (35.4%)	16 (12.80%)	<0.01
Bronchiectasis	12 (14.6%)	13 (10.40%)	0.218
Previous tuberculosis	12 (14.6%)	15 (12.00%)	0.538
**Extrapulmonary diseases**			
Diabetes mellitus	10 (12.2%)	5 (4.00%)	0.082
Cardo-cerebrovascular disease	15 (18.3%)	10 (8.00%)	0.073
Organ transplantation	1 (1.2%)	2 (1.60%)	0.629
Malignant tumor	9 (11.0%)	19 (15.20%)	0.367
Immune system diseases	19 (23.2%)	13 (10.40%)	0.128
Use of corticosteroid	32 (39.0%)	15 (12.00%)	<0.01

*CPA, chronic pulmonary aspergillosis; COPD, chronic obstructive pulmonary disease.*

### Serum *Aspergillus*-Specific Antibody Levels Among the CPA, Non-CPA and Healthy Control Groups

**FIGURE 1:**
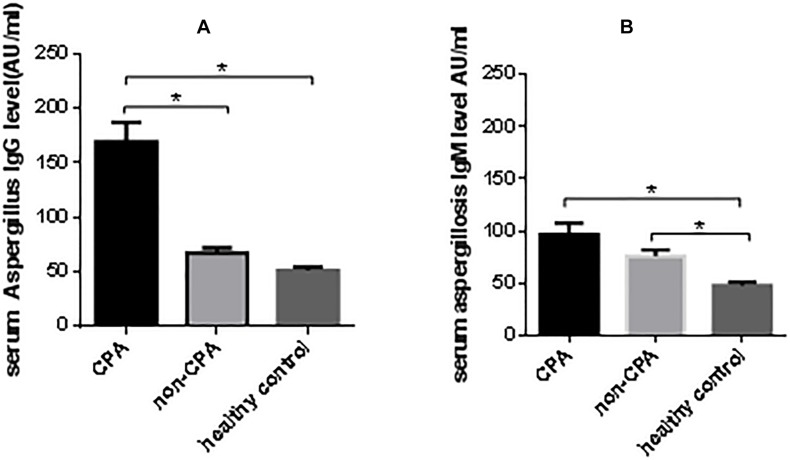
Serum aspergillus-specific antibody levels among CPA, non-CPA and healthy control group. **(A)** The mean (±SE) serum As-IgG level among CPA, non-CPA and healthy control group were 177.0 ± 21.32, 77.5 ± 8.09, and 68.7 ± 6.26 AU/ml. **(B)** The mean (±SE) serum As-IgM level among CPA, non-CPA and healthy control group were 92.3 ± 11.24, 75.5 ± 9.64, and 55.0 ± 4.34 AU/ml. ^*^P < 0.05.

The mean (±SE) serum As-IgG levels among the CPA, non-CPA and healthy control groups were 177.0 ± 21.3, 77.5 ± 8.1, and 68.7 ± 6.3 AU/ml, respectively. Using pairwise comparison, the differences in antibody levels between the CPA and non-CPA, and CPA and healthy controls were both statistically significant (*P* < 0.05), while the difference between the non-CPA and healthy controls was not (*P* = 0.29). The mean (±SE) serum As-IgM levels between the CPA, non-CPA and healthy control group were 92.3 ± 11.2, 75.5 ± 9.6, and 55.0 ± 4.3 AU/ml, respectively. Pairwise comparison revealed that the As-IgM levels in CPA and non-CPA group were significantly higher compared to levels in the healthy controls (*P* < 0.05), whereas the difference between the CPA and non-CPA group was not significant (*P* = 0.20) (Figure 1). Moreover, only 2 healthy individuals (4%) had positive As-IgG results and 3 individuals (6%) had positive As-IgM results.

### Diagnostic Performance of As-IgG, As-IgM and BALF GM

The sensitivity, specificity, NPV, PPV and false positive rate of the As-IgG test were similar to values obtained using the BALF GM test, while the false negative rate in the As-IgG test was lower than the corresponding BALF GM rate. However, the sensitivity, PPV and NPV of As-IgM were lower than respective values obtained using the BALF GM test. Interestingly, the specificity was comparable between the As-IgM and BALF GM tests. When the two antibody detection methods were combined, the sensitivity was 95.1%, specificity was 76.8%, while PPV and NPV were 72.9 and 96.0%, respectively (Table 2). When combining the results of IgG, IgM and BALF GM, the sensitivity is 96.3%, whereas the specificity is 71.2%.

**TABLE 2:** Diagnostic performance of As-IgG and As-IgM and BALF GM.

	**As-IgG**	**As-IgM**	**BALF GM (ODI ≥ 0.7)**	**Combination of As-IgG and As-IgM**
sensitivity	84.1%	43.9%	79.1%	95.1%
specificity	89.6%	87.2%	84.2%	76.8%
PPV	84.1%	69.2%	87.5%	72.9%
NPV	89.6%	70.3%	86.3%	96.0%
false positive rate	15.9%	30.7%	15.8%	23.2%
false negative rate	10.4%	29.7%	20.9%	4.0%

*As-IgG, aspergillus-specific IgG; As-IgM, aspergillus-specific IgM; BALF, bronchoalveolar lavage fluid; GM, galactomannan; ODI, optical density index; PPV, positive predictive value; NPV, negative predictive value.*

### Positive Rates of As-IgG and As-IgM Test According to the Stage of Disease

The positive rates of As-IgG among the patients with disease duration of 1–3 months (SAIA), 3–6 months, 6–9 months and ≥9 months were 87.0, 80.0, 81.8, and 80.0%, respectively. The positive rates of As-IgM among these groups were 63.0, 46.7, 0, and 0%, respectively (Table 3).

**TABLE 3:** The positive rate of As-IgG and As-IgM test among groups divided according to course of disease in CPA.

	**IgG (%)**	**IgM (%)**
<3 month (*n* = 46)	40 (87.0)	29 (63.0)
3–6 month (*n* = 15)	12 (80.0)	7 (46.7)
6–9 month (*n* = 11)	9 (81.8)	0
≥9 month (*n* = 10)	8 (80.0)	0

### Tendencies in Variation in Antibody Levels During the Course of Treatment Among CPA Patients

**FIGURE 2:**
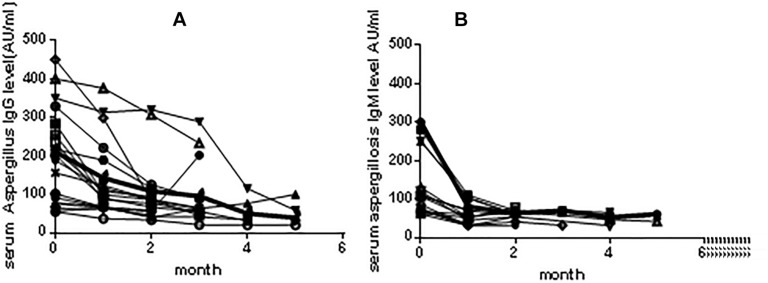
**(A)** Tendencies in variation of As-IgG levels during the course of treatment among 22 patients with CPA. There were 20 patients showed a gradual downtrend, while 2 patients showed an increasing tendency. **(B)** Tendencies in variation of As-IgM antibody levels during the course of treatment among 22 patients with CPA. Most of patients showed a gradual downtrend, while 4 patients showed elevated antibody level which didn’t above 80 AU/ml mark.

The levels of As-IgG and As-IgM in patients with CPA (*n* = 22) who had regular follow-ups were measured and the antibody variation charts were constructed (Figure 2). We noted that most patients showed a gradual decrease in As-IgG levels after 2 months of antifungal treatment. After that time frame, the levels of As-IgG continued to further decrease in all but 2 patients in whom clinical remission occurred. Those two patients showed an increasing trend in the As-IgG levels and their fungal infection was later worsened. In terms of As-IgM levels, most of the patients showed a gradual decrease, while four patients showed elevated antibody levels that did not exceed 80 AU/ml. The condition of one of those patients later aggravated.

## Discussion

Patients with CPA usually lack typical clinical and radiographic features, which makes the CPA diagnosis difficult. Early diagnosis and timely treatments are critical for reducing mortality associated with CPA. Serum GM antigen testing, which is the most widely used method, is not suitable for the CPA diagnosis because of its low sensitivity (Shin et al., 2014). The diagnosis of CPA can also be based on recovery of *Aspergillus* or positive GM in sputum samples. However, the positive rate of recovery of *Aspergillus* in CPA is quite low. Although the GM test based on sputum samples is an exploratory method for the diagnosis of pulmonary *Aspergillus* infection, there is little evidence documenting its effectiveness in the CPA diagnosis and the clinical application of sputum GM test remains controversial (Kimura et al., 2009; Fayemiwo et al., 2017). The BALF GM antigen test has an important role in early diagnosis of CPA (Zhou et al., 2017), but it requires an invasive procedure that effectively limits its application. In this study, we evaluated the utility of the As-IgG and As-IgM levels as markers for the diagnosis of CPA.

Assessment of patient population characteristics in our study revealed that CPA was more likely to occur in patients with COPD, other chronic lung diseases and in patients with a history of corticosteroid usage, which was consistent with previously published studies (De Pauw et al., 2008; Eren Akarcan et al., 2017; Garth and Steele, 2017). At first, we measured *Aspergillus*-specific antibody levels in the CPA, non-CPA and healthy control patients, and discovered that levels of both As-IgG and As-IgM were in the lower limit in healthy controls. Because BALF can only be collected using a bronchoscope, which cannot be used in patients with poor health conditions, serum As-IgG may be a more suitable screening method for the diagnosis of CPA. In addition, the sensitivity of our test (84.1%) was similar to values obtained in previous reports, which utilized Dynamiker assay (77%) and Genesis assay (75%). Additionally, our specificity (89.6%) was slightly lower compared to the previously published results obtained using Dynamiker assay (97%), Immuno CAP assay (98%), Immulite (98%), Serion (98%), Genesis (99%) and Precipitins (100%) assays (Page et al., 2016). Lower specificity in our samples can likely be explained by the fact that our control group consisted of patients with CAP, tuberculosis, bronchiectasis and lung tumors, while only healthy adults were used as controls in previously published studies. In contrast, the sensitivity of As-IgM was lower, which limited its use. However, paired measurement of As-IgG and As-IgM showed added benefit in the diagnosis of CPA over As-IgG alone (positive rate 95.1% vs. 84.1%, respectively). Reports suggest that some patients with CPA also present with hypogammaglobulinemia and appear to have a selective inability to produce *A. fumigates* IgG antibody (Denning et al., 2015). In agreement, we found that among13 patients with CPA who got negative As-IgG result in our study, two of them (15.4%) did indeed have hypogammaglobulinemia. Rimek et al. reported that IgG production starts after approximately 11 days after diagnosis of invasive aspergillosis (IA) (Kappe and Rimek, 2004), while blood IgM antibody levels can be tested for within a few days since they are typically associated with acute phase of infection (Page et al., 2015). We analyzed the positive rate of As-IgG and As-IgM based on the disease duration. Results indicated that there was no significant change in the positive rate of As-IgG with time, while the positive rate of As-IgM dropped off rapidly as the time increased.

Monitoring of the antibody levels in CPA patients undergoing antifungal treatment showed that As-IgG showed a gradual decrease in patients with remission. The levels of As-IgG increased in two patients whose condition was later worsened. These results are consistent with the previous report documenting that antibody levels could be used to monitor treatment response in patients with chronic forms of pulmonary aspergillosis (Page et al., 2015). Although most of the patients had gradually decreasing levels of As-IgM, some patients in the remission stage had elevated As-IgM levels, and this effectively reduces the use of this test in treatment response monitoring.

## Conclusion

The current work indicates that the As-IgG assay shows promising application in the diagnosis of CPA, and the As-IgG level could be used to monitor treatment response in patients with CPA. Moreover, As-IgM may have some value in the diagnosis of SAIA in patients with a relatively short disease course.

## Ethics Statement

The study protocol was approved by the Institute Ethics Committee of Jinling Hospital (2015NJKY-035-02). The study protocol conformed to the ethical guidelines of the 1975 Helsinki Declaration, and written informed consent was obtained from all subjects.

## Author Contributions

XS, HL, and YS designed the study and drafted the manuscript. HL and YR collected the samples and data, and analyzed the data. WZ, LL, and BH critically revised the manuscript. All authors involved in drafting the manuscript, and approved the final version of the manuscript.

## Conflict of Interest Statement

The authors declare that the research was conducted in the absence of any commercial or financial relationships that could be construed as a potential conflict of interest.

## Abbreviations

As-IgA: aspergillus-specific IgA
As-IgG: aspergillus-specific IgG
As-IgM: aspergillus-specific IgM
COPD: chronic obstructive pulmonary disease
CPA: chronic pulmonary aspergillosis
GM: galactomannan
NPV: negative predictive value
OD: optical density
PPV: positive predictive value.
